# Relationship Between Altered Plasma-Free Amino Acid Levels and Hyperuricemia in Dyslipidemia Without and With Hypertension

**DOI:** 10.3390/diseases12110267

**Published:** 2024-10-24

**Authors:** Rie Watanabe, M. H. Mahbub, Natsu Yamaguchi, Ryosuke Hase, Sunao Wada, Tsuyoshi Tanabe

**Affiliations:** 1 Department of Public Health and Preventive Medicine, Yamaguchi University Graduate School of Medicine, Ube 755-8505, Yamaguchi, Japan; g006ub@yamaguchi-u.ac.jp (R.W.); natsu@yamaguchi-u.ac.jp (N.Y.); hase@yamaguchi-u.ac.jp (R.H.); b106eb@yamaguchi-u.ac.jp (S.W.); tanabe@yamaguchi-u.ac.jp (T.T.); 2 Division of Systems Medicine and Informatics, Research Institute for Cell Design Medical Science, Yamaguchi University, Ube 755-8505, Yamaguchi, Japan

**Keywords:** plasma-free amino acids, hyperuricemia, dyslipidemia, hypertension, association

## Abstract

**Background:** Investigating the association between plasma-free amino acids (PFAAs) and hyperuricemia (HU) in dyslipidemia (DL) and dyslipidemia with hypertension (DH) is crucial, as it could provide valuable insights into the pathophysiology of these conditions and contribute to the development of targeted prevention and management strategies. Therefore, in this study, we aimed to elucidate the associations between PFAAs and HU in individuals with DL and DH. **Methods:** We quantified PFAAs and uric acid levels among Japanese healthy subjects (n = 1311; HU, n = 57), subjects with DL (n = 1483; HU, n = 219), and subjects with DH (n = 1159; HU, n = 237). **Results:** The concentrations of most PFAAs showed significant differences between subjects without and with HU across all groups (*p* < 0.05 to 0.001). Adjusted logistic regression analyses revealed that certain PFAAs were consistently positively or negatively associated with HU across all groups. Specifically, in the DL group, alanine, tryptophan, and tyrosine showed significant positive associations with HU, while in the DH group, citrulline and glutamate exhibited similar positive associations (*p* < 0.05 to 0.001). Conversely, threonine in the healthy group (*p* < 0.05) and glutamine in the DL group (*p* < 0.05) demonstrated significant inverse associations with HU. **Conclusions:** This study revealed a potential close relationship between alterations in PFAA profiles and HU in dyslipidemia, without and with hypertension. The findings warrant further research to elucidate the role of altered amino acid and uric acid levels as potential disease biomarkers and therapeutic targets.

## 1. Introduction

Dyslipidemia (DL), characterized by aberrant lipid levels, has emerged as a pivotal risk factor for cardiovascular disease (CVD), which accounts for a significant burden of morbidity and mortality, with a staggering annual toll of 17 million reported deaths [1,2]. The global burden of DL has escalated over the last three decades, with its prevalence reaching alarming levels for both males and females [3,4] Concurrently, hypertension has ascended to the forefront as the principal cause of CVD and premature deaths on a global scale, impacting over 1.3 billion adults [5]. Hypertension is also recognized as one of the primary comorbidities associated with DL [6]. Published cohort studies have provided strong evidence suggesting a causal relationship between DL and the subsequent development of hypertension [6,7]. Both DL, and DL with hypertension (DH), have been identified as potent synergistic risk factors for CVD [8], since the original studies from the Framingham Heart Study first published in the 1960s, emphasizing the need for a thorough understanding of their determinants and mechanisms, and necessitating closer examination of potential biomarkers to improve early detection and management.

Under homeostatic conditions, individual amino acids intricately contribute to diverse metabolic and biochemical processes within the human body [9]. Recent studies have unveiled disruptions in circulatory amino acid concentrations, particularly plasma-free amino acids (PFAAs), in individuals with lifestyle-related diseases, including DL and hypertension [10,11]. These alterations in PFAAs suggest their potential role in the pathogenesis of both DL and DH, holding promise as predictive indicators for disease development and as biomarkers for detecting and monitoring responses to therapeutic interventions. Moreover, current literature identifies hyperuricemia (HU), characterized by elevated uric acid (UA) levels, as an independent risk factor associated with DL and hypertension [12,13]. While early studies, such as those by Prior et al. in the 1960s on Polynesian populations [14], highlighted associations with high triglycerides, hypertension, and diabetes, the role of circulating amino acids in these conditions remains unknown. An intriguing perspective has been proposed, emphasizing the interplay between altered amino acid metabolism and elevated UA levels, termed the amino-uric interaction [15]. From this standpoint, it can be postulated that altered levels of amino acids, coupled with HU, may be involved in the pathogenesis of both DL and DH. Therefore, investigating the potential association between PFAAs and HU in the context of both DL and DH holds considerable significance. Uncovering such associations could offer valuable insights into disease pathophysiology, facilitating the development of targeted preventive measures, as well as diagnostic and management strategies for both conditions. Nonetheless, the intricate association between altered PFAA levels and HU in both DL and DH remains insufficiently explored, underscoring the urgency of studies to address this critical gap in current understanding. With the global burden of DL and hypertension escalating due to factors like aging and lifestyle changes, the need to elucidate the nuanced associations between PFAAs and HU in the context of both DL and DH becomes increasingly urgent.

The primary objective of this cross-sectional study was to investigate the associations between PFAAs and HU in individuals with DL and DH with adjustments for potential confounding factors. This study aimed to clarify these associations and explore their implications for understanding relevant disease mechanisms and enhancing strategies for prevention, diagnosis, and management.

## 2. Materials and Methods

### 2.1. Study Design and Population

**Figure 1:**
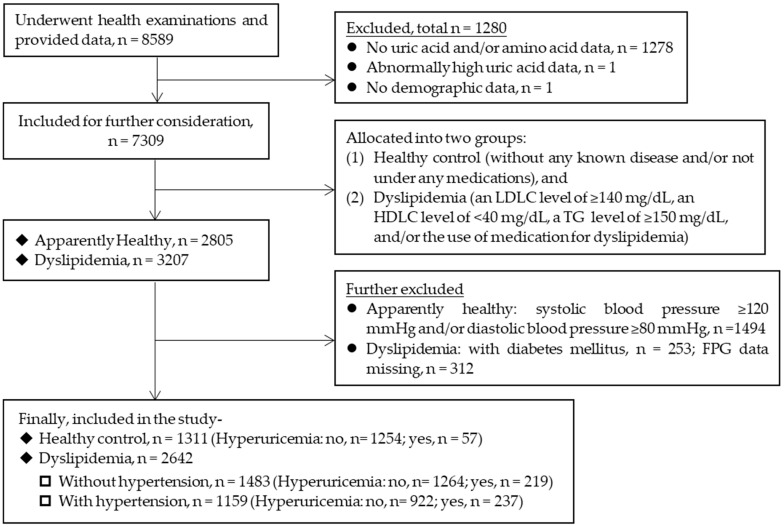
Flowchart of participants in the current study.

In this study, we conducted secondary analyses on data from a cross-sectional study involving subjects who underwent annual health check-ups between 2009 and 2011 in Shimane Prefecture, Japan, as previously reported [15]. The workflow schematic for the study is presented in Figure 1. The original study comprised 8589 participants undergoing annual health check-ups at various health examination centers. Initially, we identified a cohort of 3207 individuals diagnosed with DL from this pool. Subsequently, individuals diagnosed with diabetes mellitus (n = 253) were excluded. Diabetes was defined as having a fasting plasma glucose (FPG) level of ≥126 mg/dL, a hemoglobin A1c (HbA1c) level of ≥6.5%, and/or the use of medication(s) for diabetes mellitus. Among the remaining 2954 subjects, 312 subjects had missing FPG data and were excluded. Finally, we considered participants who were free from serious diseases (such as cancer or renal failure) or any known disorders other than the conditions applicable to the designated group, specifically dyslipidemia without and with hypertension and HU. A total of 2642 subjects with DL were included, among which 1483 subjects were included in the DL-only group and 1159 subjects in the DH group. Additionally, from the original cohort, an apparently healthy control group of 1311 subjects with systolic blood pressures (SBP) and diastolic blood pressures (DBP) of <120 mmHg and <80 mmHg, respectively, and without any known diseases was also selected. 

### 2.2. Blood Sample Collection and Storage

Venous blood samples were collected from the cubital vein of seated participants following an 8-h overnight fast. Five mL of blood was drawn into ethylenediaminetetraacetic acid tubes (Terumo, Tokyo, Japan), which were immediately placed on ice for approximately 15 min. Subsequently, the tubes underwent centrifugation at 3000 rpm for 15 min at 4 °C. Plasma was separated into tubes and stored at −80 °C for 2 weeks to 2 months until analysis of amino acids.

### 2.3. Measurement of Amino Acids and Laboratory Variables

In this study, we determined the absolute concentrations (in μmol/L) of PFAAs using a protocol described elsewhere [16]. Briefly, plasma samples were deproteinized with acetonitrile (80% final concentration), followed by pre-column derivatization with 3-aminopyridyl-N-hydroxysuccinimidyl-carbamate (APDSTAG™, Wako Pure Chemical Industries, Ltd., Osaka, Japan). Subsequently, PFAA concentrations were quantified by high-performance liquid chromatography–electrospray ionization–mass spectrometry (HPLC–ESI–MS), which allows such measurements with high accuracy. HPLC analysis was performed on a Shim-pack UF-Amino column (C18 reverse-phase column, Shimadzu Corporation, Kyoto, Japan). We assessed the absolute concentrations of 20 amino acids: Alanine (Ala), Arginine (Arg), Asparagine (Asn), Citrulline (Cit), Glutamine (Gln), Glutamate (Glu), Glycine (Gly), Histidine (His), Isoleucine (Ile), Leucine (Leu), Lysine (Lys), Methionine (Met), Ornithine (Orn), Phenylalanine (Phe), Proline (Pro), Serine (Ser), Threonine (Thr), Tryptophan (Trp), Tyrosine (Tyr), and Valine (Val).

FPG and HbA1c were measured using the hexokinase method and latex agglutination immunoassay, respectively. Enzymatic methods were employed to measure concentrations of high-density lipoprotein cholesterol (HDLC), low-density lipoprotein cholesterol (LDLC), and triglyceride (TG) in the serum. Plasma UA was quantified using the uricase-HMMPS method with the L-type UA.M kit (Wako Pure Chemical Industries, Ltd., Osaka, Japan).

### 2.4. Clinical Assessments

DL was determined by an LDLC level of ≥140 mg/dL, HDLC of <40 mg/dL, TG of ≥150 mg/dL, and/or the use of medication for dyslipidemia. Hypertension was defined as having an SBP of ≥140 mmHg, DBP of ≥90 mmHg, and/or the use of antihypertensive drugs. HU was determined as plasma UA levels of ≥7 mg/dL in men and ≥6.0 mg/dL in women, according to established literature [17,18].

On the day of the examination, trained personnel measured the resting SBP and DBP in subjects who were seated. The subjects’ arms were supported at heart level, and the measurements were taken using automated noninvasive oscillometric devices, following the recommended guidelines for this purpose [19].

### 2.5. Statistical Analyses

Continuous variables were presented as the median and interquartile range (IQR). Differences in demographic and clinical variables among groups were evaluated using the Mann-Whitney U-test for two independent samples and the Kruskal-Wallis test for k-independent samples. Bonferroni corrections were applied for multiple comparisons as appropriate. Categorical variables were analyzed using the Chi-square (χ^2^) test. We examined the association between each individual PFAAs and the outcome variable (HU) through logistic regression analyses in all groups. We adjusted the logistic regression models for only relevant significant demographic variables (first model) and for demographic and clinical variables (second model). For logistic regression analyses, all amino acids were scaled to multiples of 1 IQR for men and women separately. The results yielded odds ratios (OR) for individual amino acids, along with corresponding 95% confidence intervals (CI) and *p*-values. The data analysis was performed on anonymized data. Statistical analyses were performed using SPSS version 22 for Windows (SPSS Inc., Chicago, IL, USA). All statistical analyses were two-sided, with a significance threshold set at *p* < 0.05.

## 3. Results

The data collected from a total of 1311 healthy control subjects (463 men, 848 women) and 2642 subjects with DL (1345 men, 1297 women) were included in the final analysis of this study (Figure 1). Among the patients with DL, 1483 (751 men and 732 women) were free from hypertension, while 1159 (594 men and 565 women) had hypertension. The differences between three groups of subjects (healthy group and two groups of patients) were significant for all the demographic and clinical variables (Kruskal-Wallis Test, *p* < 0.001). Overall, compared to the patients with DL and DH, the healthy subjects were younger and had a lower BMI. Conversely, compared to the healthy subjects, both groups of patients exhibited higher values for FPG, HbA1c, LDLC, TG, SBP, DBP, and UA. In contrast, the levels of HDLC were lower in both groups of patients.

The demographic and clinical characteristics of the study populations were also compared between individuals with and without HU for each of the three groups, and presented in Table 1. The demographic variables differed significantly between the subjects without and with HU in all groups except for age in the healthy group. In both DL and DH groups, the subjects with HU were relatively younger than the subjects without HU.

On the other hand, the majority of clinical variables showed significant differences between individuals without and with HU in both DL and DH groups, with higher values generally observed in subjects with HU, except for HDLC, which was lower in this group (Mann-Whitney U-test, *p* < 0.05 to 0.001). Among healthy subjects, only TG showed a significant difference, with a higher level in those with HU compared to those without HU (Mann-Whitney U-test, *p* < 0.005).

As shown in Table 2, the concentrations of Ala, Glu, His, Ile, Leu, Lys, Phe, Pro, Tyr, and Val were significantly higher among subjects with HU in all three groups (Mann-Whitney U-test, *p* < 0.05 to 0.001). Furthermore, Met and Trp concentrations were elevated among subjects with HU in both the DL and DH groups (Mann-Whitney U-test, *p* < 0.001), while Thr concentration was higher among subjects with HU in the DH group only (Mann-Whitney U-test, *p* < 0.05). Conversely, a significant decrease in Ser concentrations was observed among subjects with HU across all three groups (Mann-Whitney U-test, *p* < 0.001). Additionally, in the DL group, significant decreases were noted in the concentrations of Cit, Gln, and Gly (Mann-Whitney U-test, *p* < 0.001) among subjects with HU, whereas among the later subjects in the DH group, significant decreases were observed in the concentrations Gln and Gly (Mann-Whitney U-test, *p* < 0.01 to 0.001).

To identify the distinct patterns of associations between PFAAs and elevated UA levels in DL without and with hypertension, we conducted binary (HU: no versus yes) logistic regression analyses after adjusting for potentially confounding demographic variables (model I), and demographic and clinical variables (model II) relevant to each group of subjects. Overall, both models showed consistent relationships of amino acids with HU in all groups (Table 3 and Table 4).

After adjusting for demographic and clinical variables (Model II), among the PFAAs, branched-chain amino acids (BCAAs, i.e., Ile, Leu, and Val) exhibited significant positive associations with HU across all three groups [OR between 1.32 and 1.68, 95% CI between 1.05 and 1.34 (lower) and 1.65 to 2.24 (upper), *p* < 0.05 to 0.001]. Furthermore, Ala and aromatic amino acids (AAAs, i.e., Phe, Trp, and Tyr) showed significant positive associations with HU in the DL group [OR between 1.23 and 1.48, 95% CI between 1.02 and 1.18 (lower) and 1.48 to 1.85 (upper), *p* < 0.05 to 0.001], while Cit, Glu, and Phe demonstrated such associations in the DH group [OR between 1.24 and 1.39, 95% CI between 1.02 and 1.16 (lower) and 1.50 to 1.67 (upper), *p* < 0.05 to 0.001]. However, the positive association of Phe with HU appeared to be stronger in the DH group compared with the DL group (*p* < 0.001 and *p* = 0.030, respectively).

Conversely, Ser showed a significant inverse association with HU across all groups [OR between 0.44 and 0.75, 95% CI between 0.28 and 0.61 (lower) and 0.69 to 0.93 (upper), *p* < 0.01 to 0.001]. Additionally, Thr in the healthy group [OR 0.66, 95% CI 0.45 (lower) and 0.96 (upper), *p* < 0.05], Gln and Gly in the DL group [OR 0.78 and 0.74, 95% CI 0.64 and 0.60 (lower) and 0.95 and 0.92 (upper), *p* < 0.05 and 0.01, respectively], and Gly in the DH group [OR 0.82, 95% CI 0.68 (lower) and 1.00 (upper), *p* < 0.05] demonstrated significant inverse associations with HU. However, the inverse association of Gly with HU appeared to be stronger in the DL group compared with the DH group (*p* < 0.01 versus *p* = 0.046).

## 4. Discussion

The results of this study shed light on the intricate relationship of altered PFAA levels with HU in dyslipidemia, both without and with hypertension, compared to apparently healthy controls. Our findings indicate distinct associations of certain PFAAs with HU that characterize both conditions.

Our study revealed elevated levels of FPG and HbA1c in dyslipidemic patients, irrespective of hypertension status. These findings align with previous research that has identified dyslipidemia as a risk factor for impaired glucose metabolism and insulin resistance [20]. Insulin resistance, often observed in DL, can lead to elevated glucose levels, which may further exacerbate lipid abnormalities, creating a vicious cycle of metabolic dysfunction. In our study, the dyslipidemic patients exhibited lower levels of HDLC, a protective lipid fraction against CVD [21]. Reduced HDLC levels in DL can compromise lipid clearance and promote atherogenic processes, further escalating cardiovascular risk.

Furthermore, our study identified higher UA levels in dyslipidemic patients compared to healthy controls. This finding is in line with existing literature as elevated levels of UA have been previously linked with metabolic syndrome, including DL, hypertension, and insulin resistance [22]. Hypertension and DL often coexist and synergistically contribute to cardiovascular morbidity and mortality [23]. Elevated blood pressure can exacerbate vascular damage initiated by DL, further increasing the risk of cardiovascular complications.

In our study, age did not differ significantly between healthy subjects without and with HU, which contrasts with the observed differences in the DL and DH groups. This finding may suggest that age-related metabolic changes played a less prominent role in the development of HU in apparently healthy individuals compared to those with DL and/or hypertension. In contrast, younger age was significantly associated with HU in both DL and DH groups, contrasting with findings from previous research linking increasing age with HU [24]. This discrepancy may suggest unique underlying mechanisms in dyslipidemic populations.

Among healthy subjects, TG was the only clinical variable the levels of which differed significantly with HU compared to those without HU. This corresponds with existing literature as elevated TG levels have been linked to increased UA production, potentially through enhanced purine metabolism [25]. On the other hand, in DL-only and DH groups, most of the clinical parameters displayed significant differences between the subjects with and without HU. These results align with previous findings suggesting that DL and hypertension are closely associated with metabolic alterations including changes in the levels of those clinical variables investigated in this study [26]. Also, the observed differences in clinical parameters between subjects with and without HU in DL and DH groups underscore the interplay between lipid metabolism and UA homeostasis [27].

In our study, individuals with HU demonstrated increased concentrations of several amino acids and decreased concentrations of others across the three examined groups. These findings align with previous research indicating alterations in multiple amino acid concentrations under conditions of HU [14,28,29,30]. Considering the observed alterations in PFAA levels between subjects with and without HU, as well as findings from existing literature, it is reasonable to postulate that the presence of HU is associated with elevated levels of specific amino acids and reduced levels of others. In this study, we identified distinct trends in PFAA changes in relation to elevated UA levels across the three groups examined. The associations between PFAAs and HU across these groups have been further discussed below, consistent with the results of logistic regression analyses adjusted for potential confounding demographic and clinical variables.

In the current study, the BCAAs (Ile, Leu, and Val) exhibited significant positive associations with HU across all three groups. Our findings are consistent with those of Kaplan et al., who found significantly elevated levels of serum BCAAs in hyperuricemic subjects [28]. BCAAs have been linked to insulin resistance and impaired glucose metabolism, which can lead to hyperinsulinemia—a known contributor to dyslipidemia and the development of hypertension [31,32,33]. This metabolic disruption may create a vicious cycle that promotes the progression of these conditions. On the other hand, published literature has shown that in DL, serum UA levels are associated with its components; specifically, total cholesterol, TG, and LDLC levels exhibit positive associations, while serum HDLC levels are inversely related [34]. Siomkajło et al. discovered significant positive correlations between plasma BCAAs and both SBP and DBP [35]. Existing evidence also indicates a distinct association between HU and hypertension, as HU can induce endothelial dysfunction, impair nitric oxide (NO) production, and promote vascular smooth muscle cell proliferation and arterial stiffening [36]. Our findings suggest a potential interactive relationship between altered levels of BCAAs with HU and other PFAAs in the development of DL and hypertension.

We observed significant positive associations of all three AAAs with HU in the DL group. These altered levels of AAAs and UA are recognized to play roles in inflammation and oxidative stress, both of which can contribute to the development of DL [31,37,38]. In contrast, in the DH group, plasma Phe showed a highly significant (positive) association with HU. Our findings align with those of Poggiogalle et al. (2019), who observed that plasma Phe, along with BCAAs, formed a cluster exhibiting a positive association with both SBP and DBP, whereas the association was not observed when considering the cluster containing the two remaining AAAs [39]. On the other hand, the significant positive association of Ala with HU in the DL group highlights its importance in DL without hypertension. Ala, a gluconeogenic amino acid, has been linked to DL and insulin resistance [40,41]. Its elevation may reflect altered gluconeogenesis and metabolic dysregulation characteristic of DL.

Ser, an amino acid with anti-inflammatory properties, plays a crucial role in various metabolic pathways and is associated with improved insulin sensitivity and lipid metabolism [42,43]. Its inverse relationship with HU across all groups as observed in our study suggests a protective role, potentially mitigating the pro-inflammatory and oxidative stress pathways that contribute to the pathogenesis of DL and hypertension. Furthermore, Thr in the healthy group, and Gln and Gly in the DL-only group, demonstrated significant inverse associations with HU. Thr can be metabolized into various important products (Gly, acetyl CoA, and pyruvate) playing a crucial role in the modulation of host metabolism and gut homeostasis [44], while Gln and Gly have been linked to anti-inflammatory and antioxidative properties [45,46], potentially explaining their inverse associations with HU.

In the DH group, Cit and Glu exhibited significant positive associations with HU. Cit is involved in the urea cycle and NO production, which plays a critical role in vascular health and blood pressure regulation [47]. Elevated Cit levels in patients with HU may indicate a compensatory response to endothelial dysfunction and reduced NO bioavailability, both of which are common in hypertension. Similarly, increased Glu levels could reflect enhanced oxidative stress, contributing to endothelial damage leading to an increase in both SBP and DBP and the progression of cardiovascular disease in hyperuricemic individuals with hypertension [48,49].

Taking all the observed findings together, this study contributes novel insights by exploring the associations between PFAAs and HU in a stratified population of individuals with DL and DH. Unlike previous studies, which often analyzed these conditions in isolation, our research highlights the nuanced interplay between PFAAs and uric acid in these prevalent health disorders. By stratifying the population into specific groups, we have uncovered distinct patterns of amino acid alterations that correlate with HU in the context of DL and DH. More research is required to fully understand these associations, as they may reveal potential pathways for targeted interventions that modulate PFAA levels to manage or prevent HU, particularly in individuals with DL and DH.

The interpretation of our study findings necessitates an acknowledgment of several potential limitations. Firstly, we did not collect data on background factors such as diet, physical activity, and alcohol consumption. While we believe the impact of these factors on our results was likely minimal due to the inclusion of a control group without DL and the uniform socio-demographic characteristics across all study groups, the absence of detailed dietary data seems to be a notable limitation. As demonstrated by the Seven Countries Study and the NI-HON-SAN Study [50,51], dietary factors—particularly fat and salt intake—play a significant role in influencing blood lipid levels and blood pressure, highlighting the importance of dietary patterns in the management of dyslipidemia and hypertension. Future studies should incorporate comprehensive nutritional data to more accurately assess how these dietary components impact PFAAs and uric acid concentrations and to better understand their role in the pathophysiology of DL and DH. Secondly, we did not stratify the results by sex. Nonetheless, we addressed this variable in our logistic regression analyses, along with other potential confounders. Additionally, we applied distinct criteria for diagnosing HU in men and women. Thirdly, the generalizability of our findings is somewhat restricted as our study was conducted exclusively among the Japanese population. Lastly, the cross-sectional design precludes any speculation regarding causality or temporal relationships between altered PFAA levels and HU in DL with or without hypertension.

## 5. Conclusions

Our study provides evidence of distinct associations between altered PFAA profiles and the presence of HU in individuals with DL, both without and with hypertension, after adjusting for potential confounders. These associations offer insights into the pathophysiological mechanisms linking specific PFAAs and HU in the development of DL or DH. Further research is needed to unravel these complex mechanisms and explore their potential as disease biomarkers and therapeutic targets.

## Figures and Tables

**Table 1:** Demographic and clinical characteristics of the study participants. Continuous variables are presented as median and IQR, while the categorical variable is reported as number (n) and percentage (%).

	Healthy					DL					DH				
	No HU		With HU			No HU		With HU			No HU		With HU		
	(n = 1254)		(n = 57)			(n = 1264)		(n = 219)			(n = 922)		(n = 237)		
Variables	Median or n	IQR or %	Median or n	IQR or %	*p*-Value	Median or n	IQR or %	Median or n	IQR or %	*p*-Value	Median or n	IQR or %	Median or n	IQR or %	*p*-Value
Sex															
Males	423	32.3	40	3.1	<0.001	579	39.0	172	11.6	<0.001	422	36.4	172	14.8	<0.001
Females	831	63.3	17	1.3		685	46.2	47	3.2		500	43.2	65	5.6	
Age (Years)	40.0	21.0	40.0	17.5	0.873	55.0	22.0	44.0	23.0	<0.001	65.0	16.0	59.0	20.5	<0.001
BMI (kg/m^2^)	20.7	3.2	22.5	3.4	<0.001	22.6	3.7	24.5	3.6	<0.001	23.8	4.0	24.7	4.9	<0.001
FPG (mg/dL)	89.0	9.0	89.0	10.5	0.361	93.0	11.8	95.0	10.0	0.006	97.0	13.0	99.0	11.5	0.154
HbA1c (%)	5.4	0.5	5.4	0.4	0.977	5.6	0.5	5.6	0.4	0.185	5.7	0.5	5.7	0.6	0.026
HDLC (mg/dL)	69.0	20.0	67.0	24.5	0.063	60.0	22.0	53.0	20.0	<0.001	59.0	20.0	53.0	18.0	<0.001
LDLC (mg/dL)	104.0	31.0	110.0	34.0	0.440	147.0	33.0	145.0	42.0	0.614	136.0	46.0	136.0	53.0	0.411
TG (mg/dL)	58.0	32.0	74.0	36.0	<0.001	99.0	74.0	161.0	113.0	<0.001	107.0	76.3	154.0	96.6	<0.001
SBP (mmHg)	110.0	11.0	110.0	9.0	0.712	122.0	15.0	123.0	13.0	0.290	140.5	17.0	142.0	17.0	0.220
DBP (mmHg)	68.0	10.0	69.0	11.0	0.700	76.0	11.0	79.0	10.0	<0.001	86.0	14.0	90.0	14.5	<0.001
UA (mg/dL)	4.3	1.6	7.1	0.9	<0.001	4.9	1.6	7.3	0.8	<0.001	5.0	1.6	7.4	0.9	<0.001

DL, dyslipidemia without hypertension; DH, dyslipidemia with hypertension; HU, hyperuricemia. BMI, body mass index; DBP, diastolic blood pressure; FPG, fasting plasma glucose; HbA1c, hemoglobin A1c; HDLC, high-density lipoprotein cholesterol; LDLC, low-density lipoprotein cholesterol; SBP, systolic blood pressure; TG, triglyceride; UA, uric acid; IQR, interquartile range. *p*-values indicate the differences, determined by a two-tailed Mann-Whitney U-test (except for ‘Sex’ determined by the χ^2^ test), between subjects without and with HU within each group.

**Table 2:** Plasma-free amino acid concentrations (μmol/L) in the study groups. Data are presented as median and interquartile range.

	Healthy					DL					DH				
	No HU		With HU			No HU		With HU			No HU		With HU		
Amino Acids	(n = 1254)		(n = 57)			(n = 1264)		(n = 219)			(n = 922)		(n = 237)		
	Median	IQR	Median	IQR	*p*-Value	Median	IQR	Median	IQR	*p*-Value	Median	IQR	Median	IQR	*p*-Value
Ala	297.5	89.3	318.2	87.9	0.043	331.8	105.7	390.2	105.2	<0.001	349.9	110.0	380.0	99.3	<0.001
Arg	89.7	25.1	88.9	28.3	0.708	96.0	21.6	97.3	22.2	0.586	96.2	23.1	98.6	21.5	0.196
Asn	44.7	9.7	45.5	7.3	0.546	45.2	8.1	45.9	8.4	0.495	44.6	8.5	45.1	7.8	0.176
Cit	26.9	8.5	26.7	9.3	0.328	29.6	8.5	27.4	7.9	<0.001	31.3	9.5	30.2	11.1	0.433
Gln	580.5	93.3	574.1	90.9	0.998	603.1	78.1	589.5	88.9	<0.001	601.4	86.3	589.8	96.6	0.005
Glu	15.5	10.4	22.9	13.6	<0.001	22.4	14.3	30.4	16.4	<0.001	24.5	14.4	31.2	16.2	<0.001
Gly	220.9	64.0	212.1	56.2	0.089	212.7	63.2	195.0	42.3	<0.001	202.8	59.0	189.1	47.3	<0.001
His	76.9	12.3	79.6	13.1	0.030	79.6	11.9	84.6	13.0	<0.001	78.6	12.2	82.4	13.2	<0.001
Ile	49.5	14.3	59.5	15.6	<0.001	55.2	18.2	66.8	18.4	<0.001	56.5	17.2	66.4	20.7	<0.001
Leu	98.8	26.6	117.6	24.4	<0.001	111.2	31.8	132.5	33.3	<0.001	110.9	32.3	126.9	31.8	<0.001
Lys	170.7	44.2	182.3	53.3	0.003	185.6	39.5	199.0	40.0	<0.001	187.9	40.8	197.8	37.2	<0.001
Met	23.3	5.9	23.9	7.0	0.097	24.3	6.1	26.4	5.6	<0.001	24.7	6.2	26.4	6.6	<0.001
Orn	42.6	14.3	45.1	8.3	0.082	47.0	12.9	45.4	13.9	0.333	49.7	12.7	50.3	12.5	0.412
Phe	52.2	9.5	55.7	7.6	<0.001	56.1	9.8	59.5	9.3	<0.001	57.6	10.9	61.0	10.2	<0.001
Pro	111.5	44.0	124.9	50.0	0.003	123.5	50.1	146.3	48.5	<0.001	125.6	49.4	141.9	47.2	<0.001
Ser	117.0	27.9	104.4	22.5	<0.001	110.5	26.1	99.8	22.4	<0.001	108.8	27.3	100.6	20.1	<0.001
Thr	120.4	35.1	117.0	29.1	0.298	119.4	32.9	122.1	25.4	0.283	120.1	34.0	123.5	35.1	0.020
Trp	49.9	11.7	54.1	14.6	0.055	53.6	12.1	58.8	11.9	<0.001	53.9	11.5	57.0	13.4	<0.001
Tyr	54.8	12.9	59.4	15.9	0.002	60.7	13.3	66.2	13.3	<0.001	64.0	16.5	67.1	17.5	<0.001
Val	184.7	44.9	213.5	63.6	<0.001	205.3	52.4	239.8	48.6	<0.001	209.9	52.5	233.5	47.5	<0.001

DL, dyslipidemia without hypertension; DH, dyslipidemia with hypertension; HU, hyperuricemia; IQR, interquartile range. Ala, alanine; Arg, arginine; Asn, asparagine; Cit, citrulline; Gln, glutamine; Glu, glutamate; Gly, glycine; His, histidine; Ile, isoleucine; Leu, leucine; Lys, lysine; Met, methionine; Orn, ornithine; Phe, phenylalanine; Pro, proline; Ser, serine; Thr, threonine, Trp, tryptophan, Tyr, tyrosine; Val, valine. *p*-values indicate the differences, determined by a two-tailed Mann-Whitney U-test, between subjects without and with HU within each group.

**Table 3:** Logistic regression analysis for association between plasma-free amino acids and hyperuricemia in each group, with adjustments for relevant demographic confounders. Odds ratios were calculated per one interquartile range change in the concentrations of the respective amino acids.

	Healthy				DL				DH			
	(No HU, n = 1254; HU, n = 57)				(No HU, n = 1264; HU, n = 219)				(No HU, n = 922; HU, n = 237)			
	OR	95% CI		*p*-Value	OR	95% CI		*p*-Value	OR	95% CI		*p*-Value
Variables		Lower	Upper			Lower	Upper			Lower	Upper	
Ala	0.98	0.69	1.41	0.929	1.60	1.30	1.97	<0.001	1.04	0.85	1.27	0.710
Arg	0.75	0.51	1.09	0.136	0.86	0.71	1.05	0.148	0.96	0.78	1.17	0.664
Asn	0.77	0.54	1.09	0.143	0.84	0.70	1.02	0.073	0.99	0.82	1.19	0.888
Cit	1.14	0.82	1.60	0.431	0.89	0.71	1.10	0.282	1.25	1.04	1.52	0.019
Gln	0.68	0.45	1.00	0.053	0.70	0.58	0.85	<0.001	0.83	0.69	1.01	0.064
Glu	1.28	0.97	1.68	0.084	1.33	1.10	1.61	0.004	1.34	1.12	1.61	0.001
Gly	0.74	0.52	1.06	0.106	0.68	0.55	0.84	<0.001	0.76	0.63	0.92	0.005
His	0.94	0.65	1.36	0.752	1.07	0.96	1.20	0.234	1.16	0.96	1.41	0.128
Ile	1.61	1.25	2.09	<0.001	1.49	1.23	1.80	<0.001	1.44	1.18	1.76	<0.001
Leu	1.68	1.27	2.24	<0.001	1.54	1.27	1.88	<0.001	1.43	1.16	1.76	<0.001
Lys	0.99	0.69	1.40	0.941	1.09	0.92	1.30	0.323	1.03	0.85	1.24	0.798
Met	0.75	0.52	1.08	0.117	0.97	0.81	1.17	0.755	1.09	0.91	1.31	0.360
Orn	0.86	0.61	1.21	0.389	0.97	0.80	1.17	0.717	1.15	0.99	1.34	0.074
Phe	1.12	0.82	1.53	0.488	1.20	1.00	1.44	0.050	1.37	1.14	1.63	<0.001
Pro	0.92	0.68	1.26	0.610	1.15	1.00	1.32	0.059	1.13	0.95	1.35	0.161
Ser	0.44	0.29	0.68	<0.001	0.50	0.40	0.63	<0.001	0.66	0.54	0.81	<0.001
Thr	0.65	0.45	0.95	0.027	0.92	0.77	1.10	0.348	1.11	0.94	1.32	0.227
Trp	0.74	0.53	1.05	0.097	1.30	1.06	1.59	0.011	1.04	0.88	1.24	0.621
Tyr	1.08	0.77	1.51	0.643	1.33	1.10	1.61	0.004	1.14	0.93	1.40	0.198
Val	1.46	1.07	1.99	0.017	1.72	1.41	2.11	<0.001	1.36	1.10	1.68	0.005

DL, dyslipidemia without hypertension; DH, dyslipidemia with hypertension; HU, hyperuricemia; OR, odds ratio; CI, confidence interval. Ala, alanine; Arg, arginine; Asn, asparagine; Cit, citrulline; Gln, glutamine; Glu, glutamate; Gly, glycine; His, histidine; Ile, isoleucine; Leu, leucine; Lys, lysine; Met, methionine; Orn, ornithine; Phe, phenylalanine; Pro, proline; Ser, serine; Thr, threonine, Trp, tryptophan, Tyr, tyrosine; Val, valine. *p*-values indicate the differences, determined by a two-tailed Mann-Whitney U-test, between subjects without and with HU within each group. Logistic regression models were adjusted for BMI and sex in the healthy group, and for age, BMI, and sex in both the DL and DH groups.

**Table 4:** Logistic regression analysis for association between plasma-free amino acids and hyperuricemia in each group, with adjustments for relevant demographic and clinical confounders. Odds ratios were calculated per one interquartile range change in the concentrations of the respective amino acids.

	Healthy				DL				DH			
	(No HU, n = 1254; HU, n = 57)				(No HU, n = 1264; HU, n = 219)				(No HU, n = 922; HU, n = 237)			
	OR	95% CI		*p*-Value	OR	95% CI		*p*-Value	OR	95% CI		*p*-Value
Variables		Lower	Upper			Lower	Upper			Lower	Upper	
Ala	0.95	0.66	1.36	0.768	1.48	1.18	1.85	<0.001	0.97	0.79	1.19	0.777
Arg	0.75	0.52	1.09	0.136	0.93	0.76	1.13	0.437	1.03	0.83	1.27	0.801
Asn	0.77	0.54	1.11	0.161	0.89	0.74	1.07	0.227	1.03	0.85	1.24	0.797
Cit	1.14	0.81	1.61	0.437	0.97	0.78	1.21	0.766	1.25	1.02	1.52	0.031
Gln	0.69	0.46	1.02	0.066	0.78	0.64	0.95	0.012	0.94	0.77	1.15	0.556
Glu	1.26	0.95	1.68	0.111	1.21	0.99	1.48	0.065	1.24	1.03	1.50	0.027
Gly	0.76	0.53	1.09	0.131	0.74	0.60	0.92	0.006	0.82	0.68	1.00	0.046
His	0.91	0.62	1.33	0.630	1.05	0.94	1.18	0.387	1.08	0.89	1.32	0.441
Ile	1.62	1.25	2.10	<0.001	1.36	1.11	1.67	0.003	1.36	1.10	1.67	0.004
Leu	1.68	1.26	2.24	<0.001	1.47	1.20	1.80	<0.001	1.38	1.12	1.71	0.003
Lys	0.98	0.68	1.39	0.889	1.15	0.96	1.38	0.122	1.09	0.89	1.32	0.422
Met	0.74	0.51	1.07	0.114	0.98	0.81	1.18	0.853	1.09	0.91	1.32	0.356
Orn	0.86	0.61	1.22	0.411	1.01	0.84	1.22	0.919	1.14	0.97	1.34	0.106
Phe	1.12	0.82	1.54	0.475	1.23	1.02	1.48	0.030	1.39	1.16	1.67	<0.001
Pro	0.92	0.68	1.26	0.612	1.09	0.95	1.27	0.225	1.06	0.89	1.28	0.511
Ser	0.44	0.28	0.69	<0.001	0.57	0.45	0.71	<0.001	0.75	0.61	0.93	0.009
Thr	0.66	0.45	0.96	0.029	0.92	0.77	1.09	0.336	1.05	0.88	1.25	0.616
Trp	0.71	0.50	1.02	0.061	1.25	1.02	1.53	0.035	0.98	0.82	1.17	0.810
Tyr	1.06	0.76	1.49	0.725	1.32	1.08	1.61	0.006	1.12	0.91	1.38	0.273
Val	1.46	1.06	2.01	0.020	1.66	1.34	2.05	<0.001	1.32	1.05	1.65	0.015

DL, dyslipidemia without hypertension; DH, dyslipidemia with hypertension; HU, hyperuricemia; OR, odds ratio; CI, confidence interval. Ala, alanine; Arg, arginine; Asn, asparagine; Cit, citrulline; Gln, glutamine; Glu, glutamate; Gly, glycine; His, histidine; Ile, isoleucine; Leu, leucine; Lys, lysine; Met, methionine; Orn, ornithine; Phe, phenylalanine; Pro, proline; Ser, serine; Thr, threonine, Trp, tryptophan, Tyr, tyrosine; Val, valine. *p*-values indicate the differences, determined by a two-tailed Mann-Whitney U-test, between subjects without and with HU within each group. Logistic regression models were adjusted for BMI, sex, and TG in the healthy group; for age, BMI, sex, DBP, FPG, HDLC, and TG in the DL group; and for age, BMI, sex, DBP, HbA1c, HDLC, and TG in the DH group.

## Data Availability

The data presented in this study are available from the corresponding author upon reasonable request.

## References

[1] GBD 2017 Causes of Death Collaborators (2018). Global, regional, and national age-sex-specific mortality for 282 causes of death in 195 countries and territories, 1980–2017: A systematic analysis for the Global Burden of Disease Study 2017. Lancet.

[2] Hedayatnia M., Asadi Z., Zare-Feyzabadi R., Yaghooti-Khorasani M., Ghazizadeh H., Ghaffarian-Zirak R., Nosrati-Tirkani A., Mohammadi-Bajgiran M., Rohban M., Sadabadi F. (2020). Dyslipidemia and cardiovascular disease risk among the MASHAD study population. Lipids Health Dis..

[3] Pirillo A., Casula M., Olmastroni E., Norata G.D., Catapano A.L. (2021). Global epidemiology of dyslipidaemias. Nat. Rev. Cardiol..

[4] World Health Organization The Global Health Observatory. https://www.who.int/data/gho/indicator-metadata-registry/imr-details/3236.

[5] Mills K.T., Stefanescu A., He J. (2020). The global epidemiology of hypertension. Nat. Rev. Nephrol..

[6] Otsuka T., Takada H., Nishiyama Y., Kodani E., Saiki Y., Kato K., Kawada T. (2016). Dyslipidemia and the risk of developing hypertension in a working-age male population. J. Am. Heart Assoc..

[7] Halperin R.O., Sesso H.D., Ma J., Buring J.E., Stampfer M.J., Gaziano J.M. (2006). Dyslipidemia and the risk of incident hypertension in men. Hypertension.

[8] Onat A., Hergenç G., Sari I., Türkmen S., Can G., Sansoy V. (2005). Dyslipidemic hypertension: Distinctive features and cardiovascular risk in a prospective population-based study. Am. J. Hypertens..

[9] Hu X., Guo F. (2021). Amino acid sensing in metabolic homeostasis and health. Endocr. Rev..

[10] Collantes E.E., Pineda P.M., Anon B.J., Sanchez J.P. (1990). Hyperuricemia-hyperlipemia association in the absence of obesity and alcohol abuse. Clin. Rheumatol..

[11] Yamakado M., Nagao K., Imaizumi A., Tani M., Toda A., Tanaka T., Jinzu H., Miyano H., Yamamoto H., Daimon T. (2015). Plasma free amino acid profiles predict four-year risk of developing diabetes, metabolic syndrome, dyslipidemia, and hypertension in Japanese population. Sci. Rep..

[12] Miyagami T., Yokokawa H., Fujibayashi K., Gunji T., Sasabe N., Okumura M., Iijima K., Naito T. (2017). The waist circumference-adjusted associations between hyperuricemia and other lifestyle-related diseases. Diabetol. Metab. Syndr..

[13] Stewart D.J., Langlois V., Noone D. (2019). Hyperuricemia and hypertension: Links and risks. Integr. Blood Press. Control.

[14] Prior A., Rose B.S., Harvey H.P., Davidson F. (1966). Hyperuricaemia, gout, and diabetic abnormality in Polynesian people. Lancet.

[15] Mahbub M.H., Yamaguchi N., Takahashi H., Hase R., Ishimaru Y., Sunagawa H., Amano H., Kobayashi-Miura M., Kanda H., Fujita Y. (2017). Association of plasma free amino acids with hyperuricemia in relation to diabetes mellitus, dyslipidemia, hypertension, and metabolic syndrome. Sci. Rep..

[16] Shimbo K., Oonuki T., Yahashi A., Hirayama K., Miyano H. (2009). Precolumn derivatization reagents for high-speed analysis of amines and amino acids in biological fluid using liquid chromatography/electrospray ionization tandem mass spectrometry. Rapid Commun. Mass. Spectrom..

[17] Iseki K., Ikemiya Y., Inoue T., Iseki C., Kinjo K., Takishita S. (2004). Significance of hyperuricemia as a risk factor for developing ESRD in a screened cohort. Am. J. Kidney Dis..

[18] Nagahama K., Iseki K., Inoue T., Touma T., Ikemiya Y., Takishita S. (2004). Hyperuricemia and cardiovascular risk factor clustering in a screened cohort in Okinawa, Japan. Hypertens. Res..

[19] Muntner P., Shimbo D., Carey R.M., Charleston J.B., Gaillard T., Misra S., Myers M.G., Ogedegbe G., Schwartz J.E., Townsend R.R. (2019). Measurement of blood pressure in humans: A scientific statement from the American heart association. Hypertension.

[20] Zheng S., Xu H., Zhou H., Ren X., Han T., Chen Y., Qiu H., Wu P., Zheng J., Wang L. (2017). Associations of lipid profiles with insulin resistance and β cell function in adults with normal glucose tolerance and different categories of impaired glucose regulation. PLoS ONE.

[21] Gordon T., Castelli W.P., Hjortland M.C., Kannel W.B., Dawber T.R. (1977). High density lipoprotein as a protective factor against coronary heart disease. The Framingham Study. Am. J. Med..

[22] Onat A., Uyarel H., Hergenç G., Karabulut A., Albayrak S., Sari I., Yazici M., Keleş I. (2006). Serum uric acid is a determinant of metabolic syndrome in a population-based study. Am. J. Hypertens..

[23] Lu S., Bao M.Y., Miao S.M., Zhang X., Jia Q.Q., Jing S.Q., Shan T., Wu X.H., Liu Y. (2019). Prevalence of hypertension, diabetes, and dyslipidemia, and their additive effects on myocardial infarction and stroke: A cross-sectional study in Nanjing, China. Ann. Transl. Med..

[24] Kuzuya M., Ando F., Iguchi A., Shimokata H. (2002). Effect of aging on serum uric acid levels: Longitudinal changes in a large Japanese population group. J. Gerontol. A Biol. Sci. Med. Sci..

[25] Hou Y.L., Yang X.L., Wang C.X., Zhi L.X., Yang M.J., You C.G. (2019). Hypertriglyceridemia and hyperuricemia: A retrospective study of urban residents. Lipids Health Dis..

[26] Nguyen N.T., Magno C.P., Lane K.T., Hinojosa M.W., Lane J.S. (2008). Association of hypertension, diabetes, dyslipidemia, and metabolic syndrome with obesity: Findings from the National Health and Nutrition Examination Survey, 1999 to 2004. J. Am. Coll. Surg..

[27] Lima W.G., Martins-Santos M.E., Chaves V.E. (2015). Uric acid as a modulator of glucose and lipid metabolism. Biochimie.

[28] Kaplan D., Bernstein D., Wallace S.L., Halberstam D. (1965). Serum and urinary amino acids in normouricemic and hyperuricemic subjects. Ann. Intern. Med..

[29] Ling Z.N., Jiang Y.F., Ru J.N., Lu J.H., Ding B., Wu J. (2023). Amino acid metabolism in health and disease. Signal Transduct. Target. Ther..

[30] Yü T.F., Kaung C., Gutman A.B. (1970). Effect of glycine loading on plasma and urinary uric acid and amino acids in normal and gouty subjects. Am. J. Med..

[31] Flores-Guerrero J.L., Groothof D., Connelly M.A., Otvos J.D., Bakker S.J.L., Dullaart R.P.F. (2019). Concentration of branched-chain amino acids is a strong risk marker for incident hypertension. Hypertension.

[32] Newgard C.B. (2012). Interplay between lipids and branched-chain amino acids in development of insulin resistance. Cell Metab..

[33] Yu L., Zhu Q., Li Y., Song P., Zhang J. (2022). Dietary branched-chain amino acids (BCAAs) and risk of dyslipidemia in a Chinese population. Nutrients.

[34] Son M., Seo J., Yang S. (2020). Association between dyslipidemia and serum uric acid levels in Korean adults: Korea National Health and Nutrition Examination Survey 2016–2017. PLoS ONE.

[35] Siomkajło M., Rybka J., Mierzchała-Pasierb M., Gamian A., Stankiewicz-Olczyk J., Bolanowski M., Daroszewski J. (2017). Specific plasma amino acid disturbances associated with metabolic syndrome. Endocrine.

[36] Vlachopoulos C., Xaplanteris P., Vyssoulis G., Bratsas A., Baou K., Tzamou V., Aznaouridis K., Dima I., Lazaros G., Stefanadis C. (2011). Association of serum uric acid level with aortic stiffness and arterial wave reflections in newly diagnosed, never-treated hypertension. Am. J. Hypertens..

[37] Matsuda M., Shimomura I. (2013). Increased oxidative stress in obesity: Implications for metabolic syndrome, diabetes, hypertension, dyslipidemia, atherosclerosis, and cancer. Obes. Res. Clin. Pract..

[38] Würtz P., Soininen P., Kangas A.J., Rönnemaa T., Lehtimäki T., Kähönen M., Viikari J.S., Raitakari O.T., Ala-Korpela M. (2013). Branched-chain and aromatic amino acids are predictors of insulin resistance in young adults. Diabetes Care.

[39] Poggiogalle E., Fontana M., Giusti A.M., Pinto A., Iannucci G., Lenzi A., Donini L.M. (2019). Amino acids and hypertension in adults. Nutrients.

[40] Newgard C.B., An J., Bain J.R., Muehlbauer M.J., Stevens R.D., Lien L.F., Haqq A.M., Shah S.H., Arlotto M., Slentz C.A. (2009). A branched-chain amino acid-related metabolic signature that differentiates obese and lean humans and contributes to insulin resistance. Cell Metab..

[41] Tai E.S., Tan M.L., Stevens R.D., Low Y.L., Muehlbauer M.J., Goh D.L., Ilkayeva O.R., Wenner B.R., Bain J.R., Lee J.J. (2010). Insulin resistance is associated with a metabolic profile of altered protein metabolism in Chinese and Asian-Indian men. Diabetologia.

[42] He L., Ding Y., Zhou X., Li T., Yin Y. (2023). Serine signaling governs metabolic homeostasis and health. Trends Endocrinol. Metab..

[43] Holm L.J., Buschard K. (2019). L-serine: A neglected amino acid with a potential therapeutic role in diabetes. APMIS.

[44] Tang Q., Tan P., Ma N., Ma X. (2021). Physiological functions of threonine in animals: Beyond nutrition metabolism. Nutrients.

[45] Aguayo-Cerón K.A., Sánchez-Muñoz F., Gutierrez-Rojas R.A., Acevedo-Villavicencio L.N., Flores-Zarate A.V., Huang F., Giacoman-Martinez A., Villafaña S., Romero-Nava R. (2023). Glycine: The smallest anti-inflammatory micronutrient. Int. J. Mol. Sci..

[46] Nemati A., Alipanah-Moghadam R., Molazadeh L., Naghizadeh Baghi A. (2019). The effect of glutamine supplementation on oxidative stress and matrix metalloproteinase 2 and 9 after exhaustive exercise. Drug Des. Dev. Ther..

[47] Rashid J., Kumar S.S., Job K.M., Liu X., Fike C.D., Sherwin C.M.T. (2020). Therapeutic potential of citrulline as an arginine supplement: A clinical pharmacology review. Paediatr. Drugs.

[48] Li R.T., Li Y., Wang B.W., Gao X.Q., Zhang J.X., Li F., Zhang X.Y., Fang Z.Z. (2023). Relationship between plasma glutamate and cardiovascular disease risk in Chinese patients with type 2 diabetes mellitus by gender. Front. Endocrinol..

[49] Shi Z., Yuan B., Taylor A.W., Dai Y., Pan X., Gill T.K., Wittert G.A. (2011). Monosodium glutamate is related to a higher increase in blood pressure over 5 years: Findings from the Jiangsu Nutrition Study of Chinese adults. J. Hypertens..

[50] Keys A. (1970). Coronary Heart Disease in Seven Countries. Circulation.

[51] Worth R.M., Kato H., Rhoads G.G., Kagan A., Syme S.L. (1975). Epidemiologic studies of coronary heart disease and stroke in Japanese men living in Japan, Hawaii and California: Mortality. Am. J. Epidemiol..

